# Plexiform Fibromyxoma: A Rare Gastric Tumor

**DOI:** 10.1155/2017/4014565

**Published:** 2017-11-26

**Authors:** Casmir Wambura, Salim Surani

**Affiliations:** ^1^Aga Khan University, Dar es Salaam, Tanzania; ^2^Texas A&M University, College Station, TX, USA

## Abstract

Plexiform fibromyxoma is a rare and distinctive benign mesenchymal neoplasm that occurs in the gastric antrum. This tumor has a potential for misdiagnosis as gastrointestinal stromal tumor (GIST). It causes mucosa and vascular ulcerations without advancement of the tumor. Cytological bland spindle cells within a variably myxoid stroma characterize the histology of the tumor. We report the case of a 41-year-old African Tanzanian lady who presented with melena and recurrent anemia. Endoscopy and imaging studies revealed antral mass with initial suspicion of a GIST. However, immunohistochemically it turned to be a plexiform fibromyxoma. Follow-up evaluation 12 months after surgery revealed no evidence of recurrence or metastasis. This is a very uncommon tumor, which, to our knowledge, has been reported only once in Africa. The clinicians need to be aware of this rare occurrence to avoid misdiagnosis as GIST tumor.

## 1. Introduction

Plexiform fibromyxoma is a recently described gastric tumor with peculiar plexiform pattern, bland spindle cells, and a myxoid stroma invading the blood vessels [1]. The tumor almost exclusively occurs in the gastric antrum and may extend into the extra gastric soft tissues or into the duodenal bulb [2, 3]. Histologically, typical plexiform intramural growth has multiple micronodules containing paucicellular to moderately cellular myxoid to collagenous and fibromyxoid neoplastic elements [4]. A prominent plexiform capillary pattern is typically present. An extramural component includes subserosal nodule, which can be cellular and solid and can have plexiform spindle cell proliferation. The tumor cell varies from oval to spindle shape with limited atypia and mitotic activity. Frequent ulceration and mucosal and vascular invasion with no adverse significance are seen in these tumors [5]. Immunohistochemically the tumor cells are positive for *α* smooth muscle actin (SMA) and variable for CD10 and consistently negative for KIT, DOG1, CD34, Desmin, and S100 protein [1, 3, 6]. No KIT or platelet-derived growth factor receptor alpha mutations are present [2]. None of the patients developed recurrence or metastases. Plexiform fibromyxoma is a distinctive benign gastric antral neoplasm that should be separated from GIST, nerve sheath tumors, and other fibromyxoid neoplasms [7]. We hereby present a case of a 41-year-old female presenting with recurrent anemia.

## 2. Case Description

**Figure 1:**
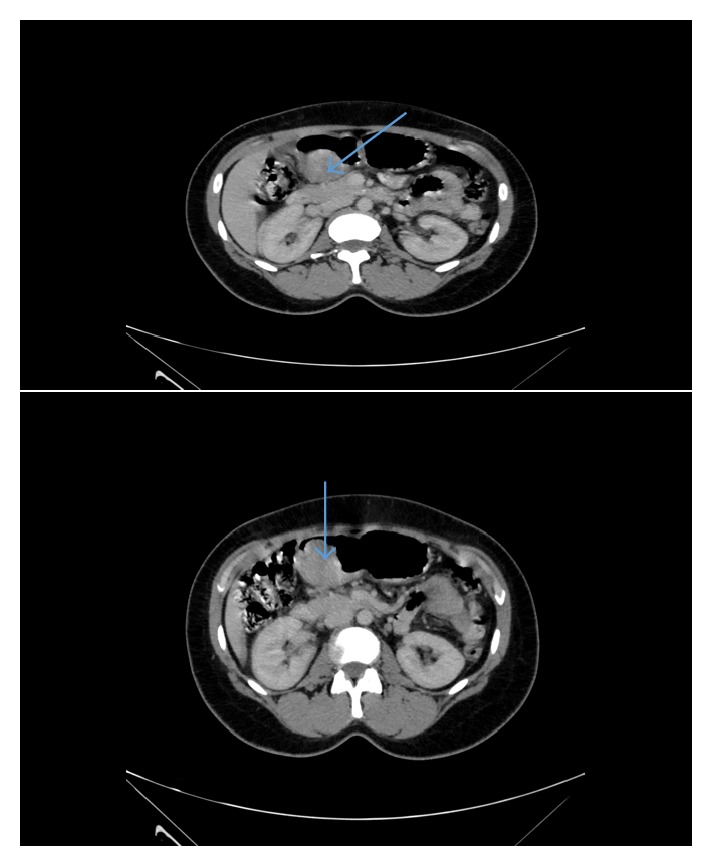
Axial images of CT scan of abdomen revealing mass in gastric antrum near pylorus.

**Figure 2:**
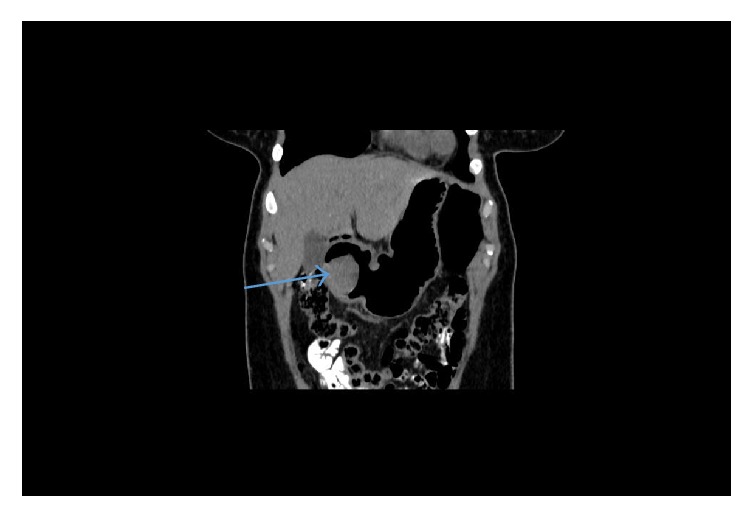
Coronal section of CT scan of abdomen, showing gastric mass near pylorus with no local invasion outside the gastric mucosa.

**Figure 3:**
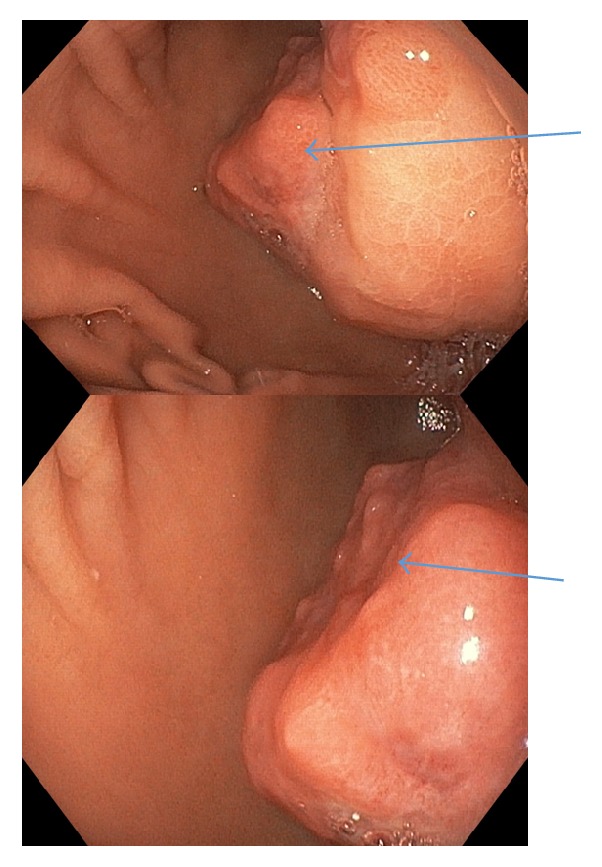
Endoscopic images showing ulcerative irregular mass emerging from the distal antrum, near posterior wall of pylorus.

**Figure 4:**
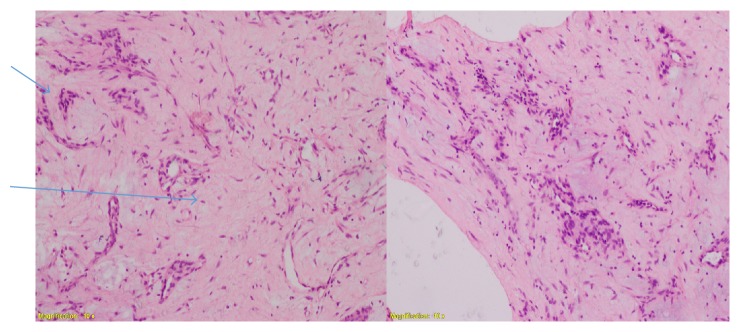
H&E stain with ×10 magnification, showing proliferation of bland myofibroblastic cells and arborizing capillaries in a loose myxoid stroma and plexiform pattern area.

A 41-year-old indigenous African Tanzanian lady was seen at gastroenterology outpatient clinic with recurrent anemia of six months' duration. She gave a history of upper abdominal discomfort associated with passing blackish tarry stools. Patient was mildly tachycardic with heart rate of 100 beats per minute. The patient's hemoglobin was 7.7 grm/dl, white blood cell count was 6.7 × 10^9^, and platelets count was 485 × 10^3^. The patient also underwent evaluation of tumor markers. The patient's carcinoembryonic antigen (CEA) was 0.96 ng/ml, CA-125 was 17.1 *μ*/ml, CA-15-3 was 9.8 *μ*/ml, and CA-19-9 showed the value of 25 *μ*/ml. The patient's alpha-fetoprotein (AFP) was 3.71 IU/ml. The patient's HIV and hepatitis screen were nonreactive. CT scan showed gastric soft mass near the pylorus with no invasiveness to the surrounding structures (Figures 1 and 2). FDG PET/CT scan revealed 4 cm × 4.5 cm endophytic mass lesion arising from the distal antrum near the pylorus of the stomach. Pedicle of the lesion was seen along the posterior-inferior wall of the antrum, likely representing primary gastric neoplasm-adenomatous polyp. Upper gastrointestinal endoscopy revealed ulcerative mass lesion with irregular surface arising from distal antrum near the posterior inferior wall of the pylorus measuring approximately 5.0 cm × 5.5 cm, most likely representing gastric neoplasm (Figure 3). The pathology of the biopsied specimen revealed proliferation of bland myofibroblastic cells and arborizing capillaries in a loose myxoid stroma. No necrosis or mitotic features were seen (Figure 4). Immunohistochemistry revealed the tumor cells, which were diffusely positive for SMA. They are negative for CD10, DOG-1, CD-117, S-100, and Desmin. The morphology and immunohistochemistry supported the diagnosis of plexiform fibromyxoma. The patient underwent distal gastrectomy plus Roux-en-Y gastrojejunostomy. Histopathology of stomach showed myxoid tumor of the stomach on frozen section. Microscopy showed submucosal plexiform growth comprising proliferating, cytological bland spindle cells and arborizing capillaries in a myxoid background. There was tumor extension through the muscularis propria into the serosa. No mitosis was noted. On immunohistochemistry tumor cells were immune-negative to CD-117 and DOG-1. The resected margins and 7 lymph nodes were free of tumor. Omentectomy margins were free of tumor. Morphology and immunohistochemistry were consistent with the diagnosis of plexiform fibromyxoma of the stomach. There was no recurrence or metastasis at the 12 months' follow-up with PET/CT scan.

## 3. Discussion

Plexiform fibromyxoma of the stomach is a novel, very rare entity of gastric mesenchymal neoplasms with nonspecific clinical manifestations. Only few cases have been reported so far since the first case described in 2007 by Takahashi et al. [1]. Until October 2016, only 19 immunohistochemically confirmed cases have been reported in the literature [3].

The plexiform fibromyxomatous gastrointestinal neoplasm has never been reported in Tanzania and East African population. In Africa, a case has been reported in a South African adult in 2010 [4]. More than 50% of the cases show erosion or ulceration of gastric mucosa, and gastrointestinal bleeding is the most common symptom. Other symptoms include upper abdominal discomfort and hematemesis. These tumors usually are excised by partial or distal gastrectomy. Plexiform fibromyxoma is a distinct benign gastric antral neoplasm, which should not be confused with a myxoid GIST [6]. This is possible by performing special immunohistochemistry demonstrating a myofibroblastic phenotype with documented expression of specific muscle actin (SMA), vimentin, Desmin, Caldesmon, and calponin processes apart from routine histology [2, 7]. Histopathology and clinical management of plexiform fibromyxoma are distinct from gastrointestinal stromal tumor (GIST). Plexiform fibromyxoma is considered to have a good prognosis with no recurrence or metastasis and should not be misdiagnosed as GIST, which requires different follow-up and treatment approaches [8].

## 4. Conclusion

The clinical presentation of plexiform fibromyxoma in terms of signs and symptoms is nonspecific. Radiological features often overlap, and upper gastrointestinal endoscopy has a limited role because of intramural location. Endoscopic ultrasound, which yields opportunity to visualize and biopsy the tumor, needs special skills. Definite diagnosis requires histological and immunohistochemical analysis. Immunohistochemical processes are not done routinely in the developing countries. The fact that plexiform fibromyxoma is a rare tumor with only a few cases in this region can lead to underrecognition and misdiagnosis of this entity and pose a real diagnosis challenge to gastroenterologists, pathologists, and surgeons when encountering such patients and differentiating plexiform fibromyxoma from other gastric intramural tumors, especially GIST.
